# Health issues and healthcare utilization among adults who reported exposure to tear gas during 2020 Portland (OR) protests: a cross-sectional survey

**DOI:** 10.1186/s12889-021-10859-w

**Published:** 2021-04-26

**Authors:** Britta N. Torgrimson-Ojerio, Karen S. Mularski, Madeline R. Peyton, Erin M. Keast, Asha Hassan, Ilya Ivlev

**Affiliations:** 1 Kaiser Permanente Northwest, Center for Health Research, 3800 N Interstate Ave, Portland, OR 97227 USA; 2 Northwest Permanente P.C, 500 NE Multnomah St #100, Portland, OR 97232 USA; 3 University of Minnesota, School of Public Health, 420 Delaware St SE, Minneapolis, MN 55455 USA

**Keywords:** Healthcare utilization, Health effects, Crowd-control, Tear gas

## Abstract

**Background:**

Repeated use of chemical irritants for crowd-control by local and federal law enforcement during sustained racial justice protests in the U.S. has raised concerns about potential adverse health effects. The objective of this study was to describe the health consequences of exposure to tear gas agents and associated healthcare utilization among adults reporting recent exposure to tear gas.

**Methods:**

A cross-sectional, self-administered web-based survey of a convenience sample of 2257 adults reporting recent exposure to tear gas in Portland, Oregon (U.S.), administered between July 30, 2020-August 20, 2020. Descriptive analyses were conducted on socioeconomic characteristics, reported health issues, utilization of healthcare services, and frequency of reported exposure to tear gas. Associations between reported mental health issues, healthcare utilization and race and/or ethnic categories were assessed using a chi-square test. For tests of association, racial and/or ethnic categories were divided into White/Non-Hispanic only and all other racial/ethnic categories due to a small number of Black, American Indian or Alaska Native, Asian/Pacific Islander, Hispanic participants and participants with multiple race and/or ethnic background. Effect sizes for the differences were expressed as Cramer’s *V*, a metric that measures associations between nominal responses. The Cochran-Armitage trend test was used to assess the relationship between health issues and the number of reported days of exposure to tear gas (i.e., a proxy dose of exposure) grouped into 1 day, 2–4 days, and ≥ 5 days. Missing data (item non-response) were omitted from the analysis.

**Results:**

Almost all respondents (2116; 93.8%) reported physical (2114; 93.7%) or psychological (1635; 72.4%) health issues experienced immediately after (2105; 93.3%) or days following (1944; 86.1%) the exposure. A slightly higher proportion experienced delayed head or gastrointestinal tract issues compared with immediate complaints. The majority (1233; 54.6%) reported receiving or planning to seek medical or mental care. We observed a positive exposure-response trend for all except mouth-related delayed issues (*p* < 0.01).

**Conclusion:**

Persons who reported exposer to tear gas agents also reported physical and psychological health issues over a multiple-day period. Health issues reported increased with the frequency of reported exposure, indicating a potential dose-response; these health effects often led to healthcare utilization. This study provides evidence of potential unexpected harms of tear gas in civilians.

**Supplementary Information:**

The online version contains supplementary material available at 10.1186/s12889-021-10859-w.

## Background

Although chemical irritants for crowd-control are increasingly used on civilians worldwide, there is no clear understanding of their potential harms on the general population [1]. Following the death of George Floyd, Jr. in May 2020 in Minneapolis, Minnesota, and the deaths of other Black Americans, instances of undue use [2] of police force, and the Black Lives Matter movement ignited ongoing protests against police brutality and systemic racism in Portland, Oregon, across the U.S. [3], and worldwide [4]. Between 15 and 26 million Americans participated [3] in nationwide protests during the first weeks of the 2020 protests. Oregon has a long history of racism, starting with sundowner laws, similar to other cities across the U.S., discriminatory policies and practices for Black persons [5]. Local and national activism surrounding inequities and calls to action have been ongoing; however, protests and crowd sizes surged upwards of thousands per night in Portland during the 100 consequtive nights of protests in 2020. As protesting crowd sizes rose, so did the use of riot control agents by law enforcement. There were single nights in Portland, OR when chemical munitions were used more than 20 times [6].

Although banned in warfare, civilians are subjected to chemical munitions when used by law enforcement agencies. Riot control agents (e.g., ortho-chloro-benzylidene-malononitrile—CS gas, 1-chloroacetophenone—CN gas, and pepper spray—oleoresin capsicum [OC]), denoted by the colloquial “tear gas,” are used to disperse crowds. These chemical agents are considered non-lethal irritants [7] and were designed to cause short-term physical discomfort to people through irritation of the eyes, nose, and respiratory system [8] and not result in severe or irreversible health effects. Most safety data on these agents come from mid-twentieth-century studies on animals and healthy young men [9]. However, health effects vary based on the type of chemicals used, proximity to deployment, and dose [10–12]. Handbooks on military medicine [13] and medical management of chemical casualties [14] suggest no serious harm to persons exposed to these chemical agents. However, data from real-world civilian exposures have documented (i) severe dermal, cardiopulmonary, ocular, and neurological injuries, (ii) permanent disabilities (e.g., blindness, loss of limbs), and even (iii) death due to respiratory arrest [8, 15, 16]. Detailed reports on healthcare utilization after tear gas exposures from emergency departments demonstrate various injuries that can result in hospitalization [12, 17]. Furthermore, concerns have been raised about the effects of police use of tear gas on mental health outcomes (e.g., depression, post-traumatic stress disorder) [18–21] and whether there is associated healthcare utilization.

Existing harms evidence in civilians appears to be insufficient to inform policy regarding the use of crowd-control agents in community settings. The short- and long-term effects of tear gas agents when used on women, children, pregnant persons, the elderly, and persons with pre-existing comorbidities, and associated utilization of healthcare remain understudied [1]. Because of reports of menstrual cycle disturbances from U.S. protesters exposed to tear gas, we sought to gather data on this phenomenon as endocrine effects of tear gas remain unstudied to date. The objective of this cross-sectional survey was to describe the health consequences of environmental exposure to tear gas agents and associated healthcare utilization among adults from the general population.

## Methods

### Study design

This was an anonymous, cross-sectional, self-administered web-based survey. The survey was open from July 30 through August 20, 2020, in English and Spanish. All responses were optional. We used the STrengthening the Reporting of OBservational studies in Epidemiology (STROBE) checklist to guide the reporting of this study.

### Participants, setting, recruitment

This survey was conducted to better understand health issues and healthcare utilization patterns in a convenience sample of adults who reported being exposed to tear gas agents in the city of Portland, Oregon. Participants were adults aged 18 or older answering two questions about the fact of and intensity of exposure to tear gas. Responses from males/cisgender men and transgender women were excluded from menstrual health outcomes questions. Participants needed access to the Internet to take the online survey.

We identified a social media site used by protesters and sought permission to post our survey, which led to social media uptake via Facebook, Twitter, Reddit, and Instagram re-postings. The web-link was later provided in a published interview by the first author in a major daily newspaper and its website (*The Oregonian*) and the Oregon Health Authority. No incentives were offered for participation.

### Variables and data sources/measurement

To develop the survey items, we adapted the Centers for Disease Control and Prevention (CDC)‘s list of known immediate and long-term health effects of exposure to riot control agents [22]. Based on anecdotal community reports, we added questions about menstrual, neurological, and mental health symptoms. Our survey included 38 questions regarding: (1) the fact of exposure to tear gas (place of exposure and the number of days exposed [i.e., proxy dose of exposure]), (2) physical and psychological health issues experienced immediately after and 1–2 days following the exposure, (3) menstrual changes, (4) utilization of healthcare services, (5) recent COVID-19 diagnosis, and (6) demographic characteristics (i.e., age, gender identity, and race and/or ethnicity). Participants were also offered an opportunity to share additional details via free text. Study data were collected and managed using Research Electronic Data Capture (REDCap) [23] tools hosted at Kaiser Permanente Center for Health Research.

### Statistical methods

Descriptive analyses were conducted on socioeconomic characteristics, reported health issues, utilization of healthcare services, and frequency of exposure to tear gas or other chemical agents. Cisgender men and transgender women were excluded from analyses of menstrual symptoms. Associations between reported mental health issues, healthcare utilization and race and/or ethnicity were assessed using chi-square tests. For tests of association, racial/ethnic categories were divided into White/Non-Hispanic only and all other racial/ethnic categories due to a smaller proportion of Black, American Indian or Alaska Native Asian/Pacific Islander, and Hispanic, respondents and persons with multiple race and/or ethnic background. Participants who did not provide race and/or ethnic information were excluded from tests of association by race. We used the chi-square test to evaluate a change in the frequency of mental health issues. Effect sizes for the differences were expressed as Cramer’s *V*, a metric that measures associations between nominal responses [24, 25]. Cramer’s *V* value of < 0.1 was considered negligible, 0.1 weak, 0.3 moderate, and 0.5 strong association. The Cochran–Armitage trend test was used to assess the relationship between health issues and number of days of exposure to tear gas (i.e., a proxy dose of exposure) grouped into 1 day, 2–4 days, and ≥ 5 days. Missing data (item non-response) were omitted from the analysis. All calculations were carried out using IBM SPSS Statistics (V.24.0) and SAS/STAT® software (V.9.4).

### Qualitative data analysis

This analysis aimed to explore qualitative responses and identify additional health concerns, context, and details not captured by the survey questions. Data were analyzed for all respondents who indicated that they had been exposed to any non-munition riot control agent, indicated the intensity of exposure, and provided answers to an open-ended question: “*Is there anything else you would like to share with us? Did you experience any other symptoms you would like to describe?*” To identify themes for this analysis, we used an inductive approach. The coding was conducted by an investigator experienced with qualitative methods.

## Results

### Participants

**Fig. 1:**
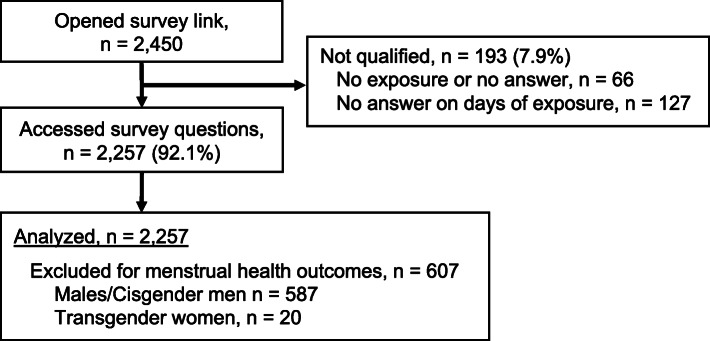
Flow-diagram

A total of 2450 persons accessed the survey (Fig. 1). Of those, 2257 (92.1%) met qualifying criteria and were eligible for our analysis. Of the 2257 persons, 1998 (88.5%) fully completed the survey. The majority self-identified as females/cisgender women (1151; 51.0% of 2257), White/non-Hispanic (1615; 71.6%), and aged 18–33 years (1199; 53.1%) (Table 1). The respondents were younger than the general population in Portland, OR. The racial and ethnic composition of the respondents was considerably close to the city population, with the exception of the proportion of persons self-identified as American Indian or Alaska Native (i.e., 4.1% among respondents vs. 0.8% in Portland, OR) and Black (2.6% among respondents vs. 5.8% in Portland, OR) [26]. The vast majority reported being exposed to tear gas at a protest (2099; 93.0%); of those, most reported two to 4 days of exposure (1391; 62.7%). A small number were exposed to tear gas only at their homes or elsewhere 158 (7.0%). Twenty-eight respondents (1.2%) reported a recent positive COVID-19 test.

**Table 1:** Participants’ characteristics

Category	n	% (***N*** = 2257)
**Age, years**		
18–33	1199	53.1
34–50	706	31.3
≥ 51	122	5.4
Prefer Not to Answer	230	10.2
**Gender Identity**		
Female/Cisgender Woman	1151	51.0
Male/Cisgender Man	587	26.0
Genderqueer, not exclusively female or male	162	7.2
Transgender woman	20	0.9
Transgender man	33	1.5
My gender identity is not listed here	27	1.2
Prefer Not to Answer	277	12.3
**Race/Ethnicity**		
White, non-Hispanic	1615	71.6
All other race/ethnicity	377	16.7
Prefer not to answer	265	11.7
**Race/Ethnicity (not mutually exclusive)**^**a**^		
White	1831	81.1
Black	58	2.6
American Indian or Alaska Native	93	4.1
Asian/Pacific Islander	115	5.1
Hispanic	174	7.7
Prefer not to answer	265	11.7
**Place of exposure to tear gas**		
At a protest	2099	93.0
Other (i.e., homes, community)	158	7.0
**Intensity of exposure in days overall**		
1	398	17.6
2 to 4	1391	61.6
≥ 5	468	20.7

^a^Respondents may be counted in multiple race/ethnicity categories

### Post-exposure health effects

Overall, 2116 (93.8%) respondents reported physical (2114; 93.7%) and/or psychological (1635; 72.4%) health issues following tear gas exposure. Besides menstrual health issues, physical health issues were reported by 2105 (93.3%) persons immediately after the exposure, abating to 1750 (77.5%) after 1–2 days (i.e., delayed health issues). Respondents more frequently reported eye, nose, mouth, skin, and/or lungs/chest issues immediately after the exposure vs. 1–2 days following (Table 2). A slightly higher proportion experienced delayed health issues related to head or gastrointestinal tract, compared with immediate complaints.

**Table 2:** Proportions of persons reporting health issues

Health issues categories	Number of persons expressing health issues^**b**^						Change from immediate to delayed issues	
	Either immediate or with a delay		Immediately		Delayed			
	n	%	n	%	n	%	n	%
Any physical or psychological health issues	2116	93.8	2105	93.3	1944	86.1	−161	−7.6
Any physical health issues^a^	2114	93.7	2105	93.3	1823	80.8	− 282	−13.4^‖^
Eyes	2001	88.7	1995	88.4	594	26.3	− 1401	−70.2^‖^
Nose	1802	79.8	1790	79.3	437	19.4	− 1353	−75.6^‖^
Mouth	1542	68.3	1510	66.9	378	16.7	− 1132	−75.0^‖^
Lungs and/or Chest	1674	74.2	1569	69.5	1063	47.1	− 506	−32.2^‖^
Skin	1312	58.1	1238	54.9	495	21.9	− 743	−60.0^‖^
Gastrointestinal	808	35.8	453	20.1	638	28.3	+ 185	+ 40.8^‖^
Head	920	40.8	610	27.0	626	27.7	+ 16	+ 2.6
Menstrual^c, d^	899	54.5	N/A		899	54.5	N/A	
Psychological health issues	1635	72.4	N/A		1635	72.4	N/A	

*N/A* not applicable

^a^ Total count for the delayed physical issues includes menstrual health/breast/chest health

^b^ Denominator was 2257 for any health issues, except 1650 for menstrual health

^c^ Excludes cisgender men/males and transgender women

^d^ For details for each menstrual/breast/chest health change, see Table 4 and Additional file 1

‖ *p* < 0.01 for the change

### Immediate physical health issues

Almost all respondents (2105; 93.3%) reported having eye, nose, lungs/chest, mouth, and/or skin-related issues immediately after the exposure (Table 2 and Additional file 1).

Of 1995 persons (88.4% of 2257) reporting any eye issues, the largest group experienced eye burning (1895; 95.0%), followed by excessive tearing (1713; 85.9%), blurred vision (1392; 69.8%), and eye redness (1078; 54.0%). Many participants reported issues related to the upper and lower respiratory tract and mouth. Of 1790 (79.3% of 2257) individuals reporting any nose-related issues, most reported having a burning sensation (1529; 85.4%) or runny nose (1520; 84.9%). Of 1569 persons (69.5%) with lung/chest issues, the largest group reported coughing (1442; 91.9%), followed by shortness of breath (1041; 66.3%), chest tightness (1038; 66.2%), and choking sensation (872; 55.6%). Mouth issues were reported by 1510 (66.9%) of respondents, most commonly irritation (1157; 76.6%), burning (1074; 71.1%), or sore throat (1064; 70.5%). Skin issues were reported by 1238 respondents (54.9%), primarily burning sensation (1189; 96.0%), rash (280; 22.6%), and burns (180; 14.5%).

Fewer individuals reported any gastrointestinal- and head-related issues immediately after the exposure (453; 20.1%). The most common gastrointestinal issues were nausea (371; 81.9% of 453) and gastrointestinal cramping (263; 58.1%). Of 610 persons reporting any head-related issues, respondents commonly cited headache (482; 79.0%), disorientation (407; 66.7%), and dizziness (356; 58.4%).

### Delayed physical health issues

Most participants (1823; 80.8%) reported delayed physical health issues after exposure (Table 2). The most commonly reported delayed issues were related to lungs and/or chest (1063; 47.1%) and menstrual changes (899; 54.5% of 1650 respondents who potentially menstruate), followed by gastrointestinal (638; 28.3%), head (626; 27.7%), and eye issues (594; 26.3%).

Delayed health issues differed in frequency when compared to those endorsed immediately after the exposure. Respondents less frequently reported eye, nose, mouth, skin, and/or lungs/chest issues days following exposure vs. immediately after the exposure (Additional file 1). For example, 1713 persons reported experiencing excessive tearing immediately; that number was 163 one to 2 days later (90.5% decline). Similar declines were seen for mouth burning (91.0% decline), drooling (96.7% decline), blurred vision (88.8% decline), eye burning (82.8% decline) nose burning (87.3% decline), and choking sensation (86.4% decline). All changes were significantly significant (*p* < 0.01).

Although the frequency of most symptoms decreased within a few days, we observed a statistically significant increase in the frequency of composit gastrointestinal issues by 40.8% (453 [20.1%] reported immediate gastrointestinal issues vs. 638 [28.3%]) reported delayed issues; *p* < 0.01). The frequency of composit head-related issues have increased by 2.6% (from 610 [27.0%] endorsed to 626 [27.7%]); however, this increase was not statistically significut 2.6% (*p* = 0.59). The most common increased gastrointestinal issues were new cases of diarrhea, gastroinstentinal cramping, and issues in the “Other” category (all *p* < 0.01). An additional 200 persons reported diarrhea—a 137.9% increase compared with the day of exposure (*p* < 0.01). Gastrointestinal cramping was reported by an additional 161 persons—a 61.2% increase (*p* < 0.01). An additional 18.5% of respondents reported having headaches one or 2 days following the exposure (*p* < 0.01).

Of 1650 persons identified as female/cisgender woman, transgender man, genderqueer (not exclusively female or male), and those who did not specify their sex or gender identity, more than half—899 (54.5%)—reported some menstrual health disruption or breast/chest tenderness (Additional file 1). The most reported issues were increased menstrual cramping in 604 (36.6%), unusual spotting in 459 (27.8%), increased bleeding in 389 (23.6%), and more days of bleeding (312; 18.9%). Respondents age 18–33 were most likely to report menstrual/breast changes (69.7%; 604 of 866). A majority of respondents age 34–50 years reported symptoms (59.2%; 271 of 458).

The frequency of any physical (except mouth-related) and/or psychological delayed health issues increased with a higher exposure to tear gas (*p* < 0.01) (Table 3).

**Table 3:** Proportions of respondents reporting health changes by intensity of exposure

Delayed health issues categories	Participants reporting health issues by intensity of exposure						Test of trend	
	1 day of exposure ***N*** = 398		2–4 days of exposure ***N*** = 1391		≥5 days of exposure ***N*** = 468			
	n	%^a^	n	%^a^	n	%^a^	***p*** value	Z value
Any physical or psychological health issues	309	77.6	1215	87.3	420	89.7	< 0.01	− 3.8
Any physical health issues^a^	290	72.9	1126	80.9	407	87	< 0.01	−4.7
Eye	91	22.9	356	25.6	147	31.4	< 0.01	−3.0
Nose	62	15.6	257	18.5	118	25.2	< 0.01	−3.8
Mouth	59	14.8	232	16.7	87	18.6	0.16	−1.4
Lungs and/or Chest	126	31.7	648	46.6	289	61.8	< 0.01	−8.3
Skin	57	14.3	294	21.1	144	30.8	< 0.01	−5.8
Gastrointestinal	77	19.3	358	25.7	203	43.4	< 0.01	−8.5
Head	91	22.9	385	27.7	150	32.1	< 0.01	−2.8
Menstrual^b^	116	39.7	566	54.8	217	66.6	< 0.01	−6.0
Psychological health issues	204	51.3	1041	74.8	390	83.3	< 0.01	−8.2

^a^ Total count for the delayed physical issues includes menstrual health/breast/chest health

^b^ Denominators for exposure of 1 day, 2–4 days, and ≥ 5 days were 292; 1032, 326, respectively, because of the eligibility criteria for this outcome (see Table 4 for details)

We also observed a positive dose-response trend for all menstrual/breast/chest health issues (*p* < 0.01), except those in the “Other” category (Table 4).

**Table 4:** Proportion of respondents reporting menstrual/breast health changes according to days of exposure

Delayed menstrual/breast/chest health issues categories	Participants reporting health issues by intensity of exposure^a^					
	1 day of exposure ***N*** = 292		2–4 days of exposure ***N*** = 1032		≥5 days of exposure ***N*** = 326	
	n	%	n	%	n	%
Any of menstrual/breast/chest health issues	116	39.7	566	54.8	217	66.6
Increased menstrual cramping	71	24.3	367	35.6	166	50.9
Unusual spotting	56	19.2	273	26.5	130	39.9
Increased bleeding	41	14.0	223	21.6	125	38.3
Increased number of days of spotting/bleeding	34	11.6	179	17.3	99	30.4
Breast/chest tenderness	23	7.9	136	13.2	74	22.7
Increased clots	9	3.1	85	8.2	66	20.2
Change in the color of blood (spotting/bleeding)	15	5.1	80	7.8	52	16.0
Absence of menstrual bleeding	9	3.1	66	6.4	32	9.8
Decreased number of days of spotting/bleeding	8	2.7	39	3.8	23	7.1
Other	14	4.8	66	6.4	23	7.1

^a^ Cochran–Armitage Trend test: *p* < 0.01 for all menstrual/breast/chest health issues, except “Other” *p* = 0.18

### Psychological health issues

Of 2257 respondents, 1635 (72.4%) reported increased anxiety, startle response, fear, fatigue, or sadness/depressive feelings after the exposure (Table 3 and Additional file 1). These issues were more common among protesters (73.7%; 1546 of 2099) than those otherwise exposed (56.3%, 89 of 158). Respondents who self-identified as persons of Black, American Indian/Alaska Native, Native Hawaiian/Pacific Islander, Asian or Hispanic race and/or ethnicity compared to White/non-Hispanic were equally likely to report mental health issues (81.4%; 307 of 377 vs. 79.0%; 1276 of 1615; *p* = 0.29, negligible association Cramer’s *V* = 0.02).

### Open-ended responses

Overall, open-ended responses align with observed quantitative results; however, these provide insights into the duration of health issues and their severity. Additional health issues identified were sleep disturbances (e.g., insomnia, nightmares), prolonged fatigue, appetite suppression, and smell and taste disturbances.

Of the 923 respondents who provided open-ended feedback, 71 commented on new headaches that were severe and lasted for weeks in some. Forty-one commented on being nauseous for days. Thirteen persons reported having diarrhea, with blood in some persons, ranging from 1 day to over a month. Overall, open-ended responses on menstrual health changes from 163 persons align with the quantitative data. Many respondents noted that after their exposures, their menstrual cycle started days or weeks earlier or later and lasted longer, compared to their typical cycle. Seventy-three persons commented on being exhausted and unable to carry out their regular work for up to 5 days; a few wrote that fatigue lasted up to 2 weeks.

Thirty-two persons indicated worsening of an existing health condition after being exposed to tear gas. Conditions and symptoms included allergies, asthma attacks that required multiple or sustained treatments or symptoms that were not resolved with treatment, flares of eczema, fibromyalgia, Hashimoto’s thyroiditis, rheumatoid arthritis, and herpes simplex virus. Sixteen persons reported injuries from projectiles (bruising, swelling, broken skin), two of which required staples or stitches.

### Healthcare utilization

The majority (1233; 54.6%) of respondents reported receiving or planning to seek medical or mental healthcare for their tear gas-related health issues.

Receiving formal medical care (i.e., care provided by a clinician) or informal medical help (i.e., care provided by a non-clinician or a person with unknown training) was reported immediately after the exposure by 41.8% (944 of 2257) of respondents. Volunteer medics were the most commonly reported immediate providers of medical attention after exposure (504; 53.4% of 944 receiving immediate care). Forty-one percent (383 of 944) reported getting medical attention from a protester who identified as a medic, nurse, doctor, or other healthcare providers. A similar proportion of individuals (421; 44.6%) reported receiving medical help from a protester who did not self-identify as a trained health provider, and six (0.6%) respondents reported care at an onsite medical utility vehicle.

Six percent (136) reported receiving professional medical care with some delay after the exposure. Of those, 69 (50.7%) reported visiting a health provider in-person or via telehealth, 30 (22.0%) called an advice nurse, 20 (14.7%) visited urgent care, and 14 (10.3%) visited an emergency room. An additional 84 (3.7%) persons reported planning to seek professional medical care.

Overall, 373 respondents (16.5%) reported receiving mental healthcare following exposure to tear gas agents, and 249 (11.0%) planned to seek mental care. We observed that a slightly higher proportion of respondents exposed at a protest reported receiving mental healthcare than those who were exposed other ways (355 [16.9%] vs. 18 [11.4%]; *p* = 0.07, negligible association Cramer’s *V* = 0.04). Among respondents exposed other ways, persons of Black, American Indian or Alaska Native, Native Hawaiian or Pacific Islander, Asian, or Hispanic race and/or ethnicity were twice as likely to report receiving mental care as White/non-Hispanic respondents (6 [28.6%] vs. 12 [12.5%]; *p* = 0.09, weak association Cramer’s *V* = 0.18). This difference did not reach statistical significance.

## Discussion

To our knowledge, this is the first large U.S. study that describes psychological and physical health issues and associated healthcare utilization by protesters and community-dwelling adults exposed to crowd-control agents. This cross-sectional study demonstrates that exposed persons commonly experienced health issues affecting multiple body systems, sometimes persisting for days to weeks and often requiring medical attention.

In the Portland, Oregon community, survey respondents reported typical health effects immediately following exposure, such as tearing and burning eyes, irritation of the upper and lower respiratory tract, and a burning sensation on the skin. These findings align with findings in small, retrospective studies conducted outside the U.S. [27–30]. In addition, respondents commonly reported delayed headaches, gastrointestinal issues, and menstrual changes that have not yet been reported in the peer-reviewed literature [8].

In our study, the majority (1635; 72.4%) of respondents reported new mental health problems. Previously, it has been demonstrated that active or indirect participation in collective actions (e.g., protests) might result in a substantial increase in prevalence of anxiety symptoms, symptoms of major depression, and post-traumatic stress disorder [31].

We acknowledge that the high level of stress and anxiety experienced by our respondents may have been a contributing factor to the physical health issues they reported and sought care for. In terms of menstrual disruption, a definitive relationship between psychological stress and menstrual changes has not been established. While some research has shown delayed or missing menstrual cycles [32] and dysmenorrhea associated with stress [33], other studies, including the California Women’s Reproductive Health Study, found no association between dysmenorrhea, hypomenorrhea, menorrhagia associated and stressful life events, though shorter cycle length and some cycle irregularities were observed [34, 35]. While distress is a plausible contributor to the menstrual disruption reported in our study, we observed high rates of persons with early onset of menses, heavier menses, and an apparent dose-response association between tear gas exposure and spontaneous bleeding and breast/chest tenderness which has not been previously reported in the peer-reviewed literature. We found no studies of the endocrine effects of tear gas agents on humans; however, toxicological studies in animals suggest an association between CS gas and changes in the endocrine system [36, 37]. It is unclear if these effects from CS exposure in laboratory studies would be observed in humans. It also remains unknown which chemical agents were used against protesters in Portland. There remains the possibility that tear gas agents have endocrine-disrupting activity. The widespread anecdotal and lay media reports of menstrual changes by protesters after exposure to tear gas agents and in this study may warrant further research on potential endocrine effects of tear gas agents.

A key review on tear gas reported 9261 injuries in 5131 persons exposed to chemical irritants used for crowd control. Almost 9 % of these injuries required professional medical management [8], although healthcare utilization was not captured comprehensively in the included studies. Our research indicates that the previously identified rate of healthcare utilization may be underestimated.

### Study considerations

Major strengths of this study are its large sample size and the fact that we surveyed adults exposed to tear gas in different settings (i.e., at a protest or in their own neighborhoods). Additionally, we collected reports of both immediate and delayed health issues and identified several newly described health issues.

While the results of this survey paint a portrait of the potential effects of exposure to tear gas in a civilian population during a specified time window, there are limits to generalizability. This study was based on a cross-sectional, self-administered web-based survey. Self-reporting in this study is subject to recall bias. The rate of reported health effects may be overestimated due to the self-selected sample and reporting exposure to tear gas. We have not captured the severity of reported health issues, nor objectively assessed participants reporting symptoms. Finally, confounding factors (e.g., stress, anxiety) were present. Exposure to chemical agents at protests was inherently associated with exposure to other law enforcement tactics, including physical force, flash-bang grenades, rubber bullets, paintball guns, and long-range acoustical devices. The last limitation is that we did not use a correction for multiple comparisons. This could potentially lead to overestimating a statistical significance of some findings.

### Future research

Further research on health consequences of exposure to chemical riot control agents is warranted. Studies should explore potential causal relationships between tear gas exposure and important health outcomes. Remaining research questions concern health effects in various populations—pregnant persons, newborns, children and adolescents, older adults, those with chronic conditions, and persons with social risk factors. Additionally, based on the menstrual health data presented here, is essential to determine if these agents can act as endocrine disruptors with the potential to impact reproductive health. Finally, the social injustice that prompted the 2020 Portland, OR protests—systemic racism and police brutality—and the violent interactions between protesters and law enforcement are factors that could contribute to immediate or sustained, novel mental health impacts in communities, which should be further explored.

### Public health implications

The 1925 Geneva Protocol prohibits the use of tear gas agents during warfare, whereas the 1997 Chemical Weapons Convention explicitly permits use for certain law enforcement purposes.

There is growing attention to policy surrounding restrictions of these chemicals in the U.S.; as of August 2020, lawmakers in nine states, including Oregon [38], had introduced bills that would ban or limit the use of tear gas by police. Our findings may further inform policy on use of these chemical agents by law enforcement in the U.S. on general populations.

## Conclusions

The short- and long-term effects of tear gas agents when used on women, children, pregnant persons, the elderly, and persons with pre-existing comorbidities and associated utilization of healthcare remain insufficient to inform policy. Our study demonstrates that community residents and protesters who reported being exposed to tear gas also commonly experienced health issues affecting multiple body systems, including previously undescribed menstrual disruption, sometimes persisting for days to weeks, and often requiring medical attention. The frequency of health issues reported increased with the exposure, indicating a potential dose-response.

## Supplementary Information

**Additional file 1.** Proportions of persons reporting health issues, detailed report.

## Data Availability

The datasets generated and/or analyzed during the current study are not publicly available but are available from the corresponding author on reasonable request.

## Abbreviations

COVID-19: Coronavirus Disease 2019
OR: Oregon (U.S. state)
