# Supplementary material for: Health issues and healthcare utilization among adults who reported exposure to tear gas during 2020 Portland (OR) protests: a cross-sectional survey
Source: BMC Public Health. 2021 Apr 26;21:803. doi: 10.1186/s12889-021-10859-w (PMC8074355; doi:10.1186/s12889-021-10859-w)
Supplement: Supplementary file 1 — Additional file 1. Proportions of persons reporting health issues, detailed report. [file 12889_2021_10859_MOESM1_ESM.docx]

**Supplemental material**

**Health Issues and Healthcare Utilization Among Adults Who Reported Exposure to Tear Gas during 2020 Portland (OR) Protests: A Cross-Sectional Survey**

Britta N. Torgrimson-Ojerio, PhD, RN^1*^;

Karen S. Mularski, MD^2^; Madeline R. Peyton, MPH^1^; Erin M. Keast, MPH^1^; Asha Hassan, MPH^3^;

Ilya Ivlev, MD, PhD, MBI^1^

* Corresponding author: [Britta.N.Torgrimson-Ojerio@kpchr.org](mailto:Britta.N.Torgrimson-Ojerio@kpchr.org)

^1^ Kaiser Permanente Northwest, Center for Health Research;
*Address*: 3800 N Interstate Ave, Portland, OR, USA 97227

^2^ Northwest Permanente P.C.;
*Address*: 500 NE Multnomah St #100, Portland, OR, USA 97232

^3^ University of Minnesota, School of Public Health;
*Address*: 420 Delaware St SE, Minneapolis, MN, USA 55455

**Additional file. Proportions of persons reporting health issues, detailed report**

| **Health issues categories** | **Number of persons expressing health issues** | | | | | | **Change from immediate to delayed** | |
| --- | --- | --- | --- | --- | --- | --- | --- | --- |
|  | **Either immediate or with a delay** | | **Immediately** | | **Delayed** | |  |  |
|  | **N** | **%** | **n** | **%** | **n** | **%** | **n** | **%** |
| **ANY PHYSICAL SYMPTOM** | **2,114** | **93.7** | **2,105** | **93.3** | **1,823** | **80.8** | **-282** | **-13.4*** |
| **Eyes** | **2,001** | **88.7** | **1,995** | **88.4** | **594** | **26.3** | **-1,401** | **-70.2*** |
| - excessive tearing | 1,723 | 86.1 | 1,713 | 85.9 | 163 | 27.4 | -1,550 | -90.5 |
| - burning | 1,901 | 95.0 | 1,895 | 95.0 | 326 | 54.9 | -1,569 | -82.8 |
| - blurred vision | 1,402 | 70.1 | 1392 | 69.8 | 156 | 26.3 | -1,236 | -88.8 |
| - redness | 1,123 | 56.1 | 1,078 | 54.0 | 301 | 50.7 | -777 | -72.1 |
| - swelling | 514 | 25.7 | 478 | 24.0 | 140 | 23.6 | -338 | -70.7 |
| - other | 156 | 7.8 | 94 | 4.7 | 80 | 13.5 | -14 | -14.9 |
| - don’t know/not sure | 43 | 2.1 | 33 | 1.7 | 11 | 1.9 | -22 | -66.7 |
| **Nose** | **1,802** | **79.8** | **1,790** | **79.3** | **437** | **19.4** | **-1,353** | **-75.6*** |
| - running nose | 1,542 | 85.6 | 1,520 | 84.9 | 265 | 11.7 | -1,255 | -82.6 |
| - burning | 1,546 | 85.8 | 1,529 | 85.4 | 194 | 44.4 | -1,335 | -87.3 |
| - swelling | 273 | 15.1 | 244 | 13.6 | 73 | 16.7 | -171 | -70.1 |
| - loss of smell | 400 | 22.2 | 369 | 20.6 | 105 | 24.0 | -264 | -71.5 |
| - other | 160 | 8.9 | 95 | 5.3 | 88 | 20.1 | -7 | -7.4 |
| - don’t know/not sure | 49 | 2.7 | 42 | 2.3 | 10 | 2.3 | -32 | -76.2 |
| **Mouth** | **1,542** | **68.3** | **1,510** | **66.9** | **378** | **16.7** | **-1,132** | **-75.0*** |
| - burning | 1,088 | 70.6 | 1,074 | 71.1 | 97 | 25.7 | -977 | -91.0 |
| - irritation | 1,182 | 76.7 | 1,157 | 76.6 | 205 | 54.2 | -952 | -82.3 |
| - sore throat | 1,111 | 72.0 | 1,064 | 70.5 | 304 | 80.4 | -760 | -71.4 |
| - difficulty swallowing | 552 | 35.8 | 528 | 35.0 | 104 | 27.5 | -424 | -80.3 |
| - drooling | 514 | 33.3 | 509 | 33.7 | 17 | 4.5 | -492 | -96.7 |
| - other | 111 | 7.2 | 86 | 5.7 | 40 | 10.6 | -46 | -53.5 |
| - don’t know/not sure | 37 | 2.4 | 31 | 2.1 | 6 | 1.6 | -25 | -80.6 |
| **Lungs and/or Chest** | **1,674** | **74.2** | **1,569** | **69.5** | **1,063** | **47.1** | **-506** | **-32.2*** |
| - coughing | 1,520 | 90.8 | 1,442 | 91.9 | 767 | 72.2 | -675 | -46.8 |
| - chest tightness | 1,175 | 70.2 | 1,038 | 66.2 | 691 | 30.6 | -347 | -33.4 |
| - choking sensation | 890 | 53.2 | 872 | 55.6 | 119 | 11.2 | -753 | -86.4 |
| - noisy breathing (wheezing) | 606 | 36.2 | 531 | 33.8 | 309 | 29.1 | -222 | -41.8 |
| - shortness of breath | 1,141 | 68.2 | 1041 | 66.3 | 574 | 54.0 | -467 | -44.9 |
| - other | 119 | 7.1 | 78 | 5.0 | 64 | 6.0 | -14 | -17.9 |
| - don’t know/not sure | 14 | 0.8 | 11 | 0.7 | 5 | 0.5 | -6 | -54.5 |
| **Skin** | **1,312** | **58.1** | **1,238** | **54.9** | **495** | **21.9** | **-743** | **-60.0*** |
| - burning sensation | 1,241 | 94.6 | 1,189 | 96.0 | 338 | 68.3 | -851 | -71.6 |
| - rash | 369 | 28.1 | 280 | 22.6 | 218 | 44.0 | -62 | -22.1 |
| - burns on skin | 207 | 15.8 | 180 | 14.5 | 92 | 18.6 | -88 | -48.9 |
| - blisters | 95 | 7.2 | 73 | 5.9 | 60 | 12.1 | -13 | -17.8 |
| - other | 94 | 7.2 | 44 | 3.6 | 63 | 12.7 | +19 | +43.2 |
| - don’t know/not sure | 23 | 1.8 | 21 | 1.7 | 3 | 0.6 | -18 | -85.7 |
| **Gastrointestinal** | **808** | **35.8** | **453** | **20.1** | **638** | **28.3** | **+185** | **+40.8*** |
| - nausea | 577 | 71.4 | 371 | 81.9 | 385 | 60.3 | +14 | +3.8 |
| - vomiting | 183 | 22.6 | 143 | 31.6 | 85 | 13.3 | -58 | -40.6 |
| - cramping | 524 | 64.9 | 263 | 58.1 | 424 | 66.5 | +161 | +61.2 |
| - diarrhea | 384 | 47.5 | 145 | 32.0 | 345 | 54.1 | +200 | +137.9 |
| - other | 105 | 13.0 | 37 | 8.2 | 87 | 13.6 | +50 | +135.1 |
| - don’t know/not sure | 9 | 1.1 | 6 | 1.3 | 4 | 0.6 | -2 | -33.3 |
| **Head** | **920** | **40.8** | **610** | **27.0** | **626** | **27.7** | **+16** | **+2.6** |
| - headache | 798 | 86.7 | 482 | 79.0 | 571 | 91.2 | +89 | +18.5 |
| - dizziness | 482 | 52.4 | 356 | 58.4 | 241 | 38.5 | -115 | -32.3 |
| - disoriented | 555 | 60.3 | 407 | 66.7 | 257 | 41.1 | -150 | -36.9 |
| - other | 32 | 5.2 | 32 | 5.2 | This was not asked on survey | | | |
| - don’t know/not sure | 21 | 2.3 | 16 | 2.6 | 5 | 0.8 | -11 | -68.8 |
| **Menstrual health** | **899** | **54.5** | **N/A** |  | **899** | **54.5** | **N/A** |  |
| - increased menstrual cramping | 604 | 36.6 |  |  | 604 | 36.6 |  |  |
| - increased bleeding | 389 | 23.6 |  |  | 389 | 23.6 |  |  |
| - absence of menstrual bleeding | 107 | 6.5 |  |  | 107 | 6.5 |  |  |
| - unusual spotting/different than your typical | 459 | 27.8 |  |  | 459 | 27.8 |  |  |
| - change in the color of blood during spotting or bleeding | 147 | 8.9 |  |  | 147 | 8.9 |  |  |
| - increase in clots | 160 | 9.7 |  |  | 160 | 9.7 |  |  |
| - increase in the number of days of spotting/bleeding | 312 | 18.9 |  |  | 312 | 18.9 |  |  |
| - decrease in the number of days of spotting/bleeding | 70 | 4.2 |  |  | 70 | 4.2 |  |  |
| - breast/chest tenderness | 233 | 14.1 |  |  | 233 | 14.1 |  |  |
| - other | 103 | 6.2 |  |  | 103 | 6.2 |  |  |
| **ANY PSYCHOLOGICAL HEALTH ISSUES** | **1,635** | **72.4** | **N/A** |  | **1,635** | **72.4** | **N/A** |  |

N/A – not applicable;* p < 0.01 for the change
